# Supplementary material for: Synthesis and Physico-Chemical Properties of Homoleptic Copper(I) Complexes with Asymmetric Ligands as a DSSC Dye
Source: Molecules. 2021 Nov 12;26(22):6835. doi: 10.3390/molecules26226835 (PMC8623343; doi:10.3390/molecules26226835)
Supplement: Supplementary file 1 [file molecules-26-06835-s001.zip › molecules-1444777-supplementary.pdf]

Supplementary Materials

Synthesis and Physico-chemical Properties of Homoleptic Copper(I) Complexes with Asymmetric Ligands as a Dye of DSSC

Tomohiko Inomata [1,*], Mayuka Hatano [1], Yuya Kawai [1], Ayaka Matsunaga [1], Takuma Kitagawa [1], Yuko Wasada-Tsutsui [1], Tomohiro Ozawa [1] and Hideki Masuda [2,*]

[1] Department of Life Science and Applied Chemistry, Graduate School of Engineering, Nagoya Institute of Technology, Gokiso-cho, Showa-ku, Nagoya 466-8555, Japan

[2] Department of Applied Chemistry, Aichi Institute of Technology, 1247 Yachigusa Yakusa-cho, Toyota 470-0392, Japan

* Correspondence: tino@nitech.ac.jp (T.I.); masuda.hideki@aitech.ac.jp (H.M.)

Contents:

Experimental. Detailed synthetic methods for **L1**, **L2**, **L3**, **M1**, **M2**, and **Y3**.

Figure S1. ^{1}H NMR spectra of a) **M1**, b) **M2**, and c) **Y3** in CD_3OD.

Figure S2. ESI-MS spectra of a) **M1**, b) **M2**, and c) **Y3** in MeOH (positive mode).

Figure S3. Cyclic voltammograms of a) **M1**, b) **M2**, and c) **Y3**. Concentration of each dye: 0.3 mM. Electrolyte solution: 0.1 M TBAP in DMF (for **M1** and **M2**) and in EtOH (for **Y3**).

Figure S4. Cyclic voltammograms of a) **M1**, b) **M2**, and c) **Y3** on the TiO_2/FTO electrode. Electrolyte solution: 0.1 M TBAP in MeCN.

Figure S5. Isosurfaces plot of molecular orbitals of **M1** in EtOH, as calculated using CAM-B3LYP.

Figure S6. Isosurfaces plot of molecular orbitals of **M2** in EtOH, as calculated using CAM-B3LYP.

Figure S7. Isosurfaces plot of molecular orbitals **Y3** in EtOH, as calculated using CAM-B3LYP.

Figure S8. Incident photo-to-current conversion efficiency spectra for dye-sensitized solar cells

Table S1. Amount of each dye adsorbed on the TiO_2/FTO electrode.

Experimental

Detailed synthetic methods for L1, L2, L3, M1, M2, and Y3.

(6,6′-Dimethyl-2,2′-bipyridine-4-yl)methanol [1]

A mixture of 6,6′-dimethyl-2,2′-bipyridine-4-carboxylate (2.07 g, 8.54 mmol) and $NaBH_4$ (405 mg, 10.7 mmol) in dry EtOH (40 mL) was refluxed under Ar for 1 day. The reaction mixture was neutralized by a saturated NH_4Cl aqueous solution. After the addition of water and extraction with CH_2Cl_2, the organic layer was dehydrated with Na_2SO_4, and concentrated to obtain a light yellow compound (1.30 g, 71.2%).

^{1}H NMR ($CDCl_3$, 300 MHz) δ/ppm: 2.63 (s, 6H), 4.77 (s, 2H), 7.16 (d, 1H), 7.18 (s, 1H), 7.69 (t, 1H), 8.12 (s, 1H), 8.17 (d, 1H).

6,6′-Dimethyl-2,2′-bipyridine-4-carbaldehyde

A mixture of (6,6′-dimethyl-2,2′-bipyridine-4-yl)methanol (517 mg, 2.41 mmol), $PhI(OAc)_2$ (1.51g, 4.69 mmol), and TEMPO (32.6 mg, 0.209 mmol) in CH_2Cl_2 was stirred for 18 h at rt. After the addition of saturated $NaHCO_3$ aqueous solution (150 mL) into the reaction mixture and the extraction by CH_2Cl_2, the organic layer was dehydrated with Na_2SO_4 and concentrated. The residue was purified by silica gel column chromatography (eluted with CH_2Cl_2/ethyl acetate = 3/1) to obtain a light orange-white solid (304 mg, 59.3%).

^{1}H NMR ($CDCl_3$, 300 MHz) δ/ppm: 2.66 (s, 3H), 2.73 (s, 3H), 7.21 (d, 1H), 7.27 (s, 1H), 7.56 (s, 1H), 7.72 (t, 1H), 8.24 (d, 1H), 8.64 (s, 1H), 10.16 (s, 1H).

(E)-Ethyl 2-cyano-3-(6,6′-dimethyl-2,2′-bipyridine-4-yl)acrylate

Ethyl 2-cyanoacetate (0.15 ml, 1.41 mmol) and 6,6′-dimethyl-2,2′-bipyridine-4-carbaldehyde (297 mg, 1.40 mmol) were dissolved in EtOH (5 mL). After the addition of a few drops of piperidine into the solution, the reaction mixture was stirred for 30 min at rt. The resultant precipitation was filtered, washed with water, and dried to obtain white powder (268 mg, 62.2%).

^{1}H NMR ($CDCl_3$, 300 MHz) δ/ppm: 1.43 (t, 3H), 2.64 (s, 3H), 2.72 (s, 3H), 4.42 (q, 2H), 7.21 (d, 1H), 7.72 (t, 1H), 7.77 (s, 1H), 8.22 (d, 1H), 8.31 (s, 1H), 8.53 (s, 1H).

Potassium (E)-2-cyano-3-(6,6′-dimethyl-2,2′-bipyridine-4-yl)acrylate (L1)

The compound was synthesized according to the literature.[2] (*E*)-Ethyl 2-cyano-3-(6,6′-dimethyl- 2,2′-bipyridine-4-yl)acrylate (155 mg, 0.50 mmol) was dissolved in

EtOH/CH_2Cl_2 (2/1). After adding 5 % w/w KOH/EtOH solution (5 ml) into the solution, the reaction mixture was refluxed at 70°C for 30 min. The suspension was cooled to rt, and the precipitate was filtered, washed with diethyl ether. The resultant precipitate was dried to obtain an yellowish-white solid. Furthermore, the solid was washed with methanol and dried to obtain white solid of the potassium salt (64.3 mg, 40.4%).

^{1}H NMR (DMSO-d_6, 300 MHz) δ/ppm: 2.57 (s, 3H), 2.60 (s, 3H), 7.32 (d, 1H), 7.60 (s, 1H), 7.83 (t, 1H), 7.86 (s, 1H), 8.18 (d, 1H), 8.59 (s, 1H). FT-IR (solid, KBr) ν/cm^{-1}: ν 3423 (O-H), 2924 (C-H), 2223 (C≡N), 1655 (C=O), 1612, 1584, 1557, 1460, 1413, 1360, 1322, 1262 (C=C, C=N), δ 796 (C-H).

Preparation of [Cu(L1)$_2$]Cl (M1)

To an aqueous solution (2 mL) of **L1** (31.7 mg, 0.1 mmol) was dissolved $CuSO_4\cdot 5H_2O$ (13.4 mg, 0.054 mmol). After the addition of a few drops of 1 M KOH aq. into the solution, the aqueous solution (2 mL) of ascorbic acid (11.2 mg, 0.064 mmol) was added to the solution to obtain the purple solution. The pH of the reaction mixture was adjusted to pH 2.0 by adding 1 M HCl, and the generated precipitate was filtered, washed with water, and dried to obtain purple powder (29.6 mg, 42.1%).

^{1}H NMR (CD_3OD, 300 MHz) δ/ppm: 2.27 (s, 6H), 2.33 (s, 6H), 7.61 (d, 2H), 7.98 (s, 1H), 8.12 (t, 2H), 8.13 (s, 2H), 8.42 (d, 2H), 8.83 (s, 1H). ESI-TOF-MS m/z: 620.99 [M-Cl]$^+$ (calc. 621.13).

FT-IR (solid, KBr) ν/cm^{-1}: ν 3233 (O–H), 2961, 2925 (C–H), 2221 (C≡N), 1703 (C=O), 1638, 1600, 1545, 1451, 1354, 1260 (C=C, C=N), δ 805 (C-H). Anal. calcd. for $C_{32}H_{26}Cl_1Cu_1N_6O_4\cdot 2.5H_2O$: C, 54.70; H, 4.45; N, 11.96. Found: C, 54.79; H, 4.60; N, 11.77.

N'-(3-(4-Dimethylamino)phenyl)allvidene)-4-methylbenzenesulfonohydrazide

p-Toluenesulfonyl hydrazide (2.05 g, 11 mmol) was added to 4-(dimethylamino)cinnamaldehyde (1.77 g, 10.1 mmol) in MeOH (60 mL), which was stirred for 2.5 h at rt. Additionally, the reaction solution was cooled overnight, and the resultant precipitate was filtered and dried to obtain a yellow solid (3.07 g, 88.5%).

^{1}H NMR ($CDCl_3$, 300 MHz) δ/ppm: 2.42 (s, 3H), 2.99 (s, 6H), 6.65 (d, 1H), 6.66 (d, 2H), 6.66 (t, 1H), 7.30 (d, 2H), 7.32 (d, 2H), 7.49 (s, 1H), 7.53 (d,1H), 7.84 (d, 2H).

N,N-Dimethyl-4-(1H-pyrazol-3-yl)aniline [3]

K_2CO_3 (3.16 g) was added to *N'*-(3-(4-dimethylamino)phenyl)allvidene)-4-methylbenzenesulfono-hydrazide (1.87 g, 5.44 mmol) in MeOH (100 mL), and the resultant solution was refluxed for 20 h. To the reaction mixture was added water (200

mL) and extracted with chloroform. The organic layer was dehydrated with Na_2SO_4 and concentrated to obtain deep yellow solid (855 mg, 84.0%).
^{1}H NMR ($CDCl_3$, 300 MHz) δ/ppm: 3.00 (s, 6H), 6.49 (d, 1H), 6.77 (2H, d), 7.57 (d, 2H), 7.59 (s, 1H).

Methyl 2-(3-(4-(dimethylamino)phenyl)-1H-pyrazol-1-yl)-6-methylisonicotinate

The suspension of NaH (264 mg, 11.0 mmol) in DMF (10 mL) was slowly added into *N,N*-dimethyl-4-(1H-pyrazol-3-yl)aniline (1.31 g, 7.02 mmol) in DMF (10 mL) under Ar. After the solution was heated at 100°C for 45 min, methyl 2-chloro-6-methylpyridine-4-carboxylate (1.32 g, 7.12 mmol) in DMF (10 mL) was added, and the resultant reaction mixture was heated at 130°C for 3 days. After the solvent was removed, water (15 mL) was added and pH of the solution was adjusted to acidity by addition of 3 M HCl. The generated precipitate was removed, and the reaction mixture was neutralized with NaOH aq., and then extracted with chloroform. The organic layer was dehydrated with Na_2SO_4 and concentrated. The residue was purified by silica gel column chromatography (eluted with $CHCl_3$/MeOH=15/1) and alumina column (CH_2Cl_2/MeOH) to obtain a flesh-colored solid (93.2 mg, 3.95%).
^{1}H NMR ($CDCl_3$, 300 MHz) δ/ppm: 2.57 (s, 3H), 3.01 (s, 6H), 3.15 (s, 3H), 6.68 (d, 1H), 6.78 (d, 2H), 7.00 (s, 1H), 7.79 (d, 2H), 7.86 (s, 1H), 8.56 (d, 1H).

2-(3-(4-(Dimethylamino)phenyl)-1H-pyrazol-1-yl)-6-methylisonicotinic acid (L2) [4]

Methyl 2-(3-(4-(dimethylamino)phenyl)-1H-pyrazol-1-yl)-6-methylisonicotinate (87.1 mg, 0.259 mmol) was dissolved in MeOH (40 mL), and K_2CO_3(460 mg, 3.33 mmol) in water (3 mL) was added into the solution. After the reaction mixture was refluxed at 80°C for 24 h, MeOH was removed. The pH of the solution was adjusted to 3.0 by adding 2 M HCl to obtain yellow solid (81.2 mg, 97.3%).
^{1}H NMR (CD_3OD, 300 MHz) δ/ppm: 2.59 (s, 1H), 2.99 (s, 6H), 6.77 (d, 1H), 6.83 (d, 2H), 7.55 (s, 1H), 7.79 (d, 2H), 8.27 (s, 1H), 8.55 (d, 1H). FT-IR (solid, KBr) ν/cm^{-1}: ν 3431 (O−H), 2923, 2813 (C−H), 1703 (C=O), 1616, 1569, 1512, 1469, 1435, 1361 (C=C, C=N), δ 766, 711 (C−H).

Preparation of [Cu(L2)$_2$]Cl (M2)

A few drops of 1 M NaOH were added into the suspension of **L2** (32.3 mg, 0.10 mmol) in water (2 mL). After the addition of $CuSO_4·5H_2O$ (13.4 mg, 0.0537 mmol) in the water (2 mL), an aqueous solution (2 mL) of ascorbic acid (17.5 mg, 0.0994 mmol) was added into the reaction mixture to obtain a yellow solution. The pH of the mixture was

adjusted to 3.0 by adding 1 M HCl aq., and then the generated precipitation was filtered, washed with water, and dried. Furthermore, the obtained solid was dissolved in EtOH to remove the insoluble compound. The solution was concentrated and dried to obtain a deep yellow powder (3.8 mg, 4.55%).

^{1}H NMR (CD_3OD, 300 MHz) δ/ppm: 2.23 (s, 6H), 2.85 (s, 12H), 6.42 (d, 4H), 7.10 (d, 2H), 7.65 (s, 2H), 7.66 (d, 4H), 8.20 (s, 2H), 8.80 (d, 2H). ESI-TOF-MS *m*/*z*: 706.85 $[M-Cl]^+$ (calc. 707.22). FT-IR (solid, KBr) ν/cm^{-1}: ν 3444 (O−H), 2804 (C−H), 1716 (C=O), 1611, 1557, 1512, 1434, 1363, 1317 (C=C, C=N), δ771, 701 (C−H). Anal. calcd. for $C_{36}H_{36}Cl_1Cu_1N_8O_4$: C, 58.14; H, 4.88; N, 15.07. Found: C, 58.27; H, 5.12; N, 14.79.

3-Cyano-6-methyl-2-oxo-1, 2-dihydro-pyridine-4-carboxylic acid ethyl ester

Na (4.5 g, 0.20 mol) was added to EtOH (100 mL) and the resultant mixture was stirred for one night. The mixture of diethyl oxalate (22.7 mL, 0.17 mmol) and acetone (12.3 mL, 0.17 mmol) was slowly added to the resultant mixture and stirred for 3 h at RT. Cyanoacetamide (14 g, 0.17 mol) was added to the resultant pale yellow suspension and stirred for 6 h at 80 °C. After removal of EtOH, H_2O (40 mL) and AcOH (3.3 mL) were added to the residue. After filtration, the resultant orange precipitation was obtained (20.7 g, 59%).

^{1}H NMR (DMSO-d_6, 300 MHz) δ/ppm: 1.31 (t, 3H), 2.32 (s, 3H), 4.35 (q, 2H), 6.55 (s, 1H).

6-Methyl-2-oxo-1,2-dihydropyridine-4-carboxylic acid

3-Cyano-6-methyl-2-oxo-1, 2-dihydro-pyridine-4-carboxylic acid ethyl ester (5g, 24 mmol) was dissolved to 6M HCl aq. (30 mL) and refluxed for 12 h. After cooling to RT, the resultant solution was neutralized with $NaHCO_3$. The beige precipitate was appeared by cooling to 0 °C. The precipitate was filtered and washed with H_2O. The precipitate was dried *in vacuo* (3.3 g, 89%).

^{1}H NMR (DMSO-d_6, 300 MHz) δ/ppm: 2.23 (s, 3H), 6.37 (s, 1H), 6.61 (s, 1H).

2-Chloro-6-methylpyridine-4-carboxylic acid methyl ester

6-Methyl-2-oxo-1,2-dihydropyridine-4-carboxylic acid (4.72 g, 31 mmol) was added to phosphoryl chloride (2 mL) and refluxed for 18 h. After removal of excess phosphoryl chloride *in vacuo*, MeOH (20 mL) was added to the residue on an ice bath. The resultant solution was refluxed for 24 h and neutralized with $NaHCO_3$. The resultant solution was extracted by H_2O and AcOEt, and the organic layer was dried over Na_2SO_4. After removal of the solvent by evaporation, the pale yellow solid was obtained. The resultant solid was purified by alumina column chromatography (eluent: CH_2Cl_2) and

white solid was obtained (3.4 g, 59%).
^{1}H NMR (DMSO-d_6, 300 MHz) δ/ppm: 2.60 (s, 3H), 3.95 (s, 3H), 7.63 (s, 1H), 7.68 (s, 1H).

2-bromo-6-methyl pyridine

2-Amino-6-methyl pyridine (10.8 g, 0.1 mol) was added to 48% HBr aq. (49.4 mL) at –20 °C. Br_2 (15 mL) was slowly added to the solution at 0 °C and the resultant reddish-black solution was obtained. The aqueous solution (26 mL) of $NaNO_3$ (17 g, 0.25 mol) was slowly added to the solution under 5 °C and stirred for 30 min. After stirring, the aqueous solution (40 mL) of NaOH (38 g) was added to the solution. The resultant yellow suspension was extracted with Et_2O. Yield 8.4 g (49%).
^{1}H NMR (DMSO-d_6, 300 MHz) δ/ppm: 2.51 (s, 3H), 6.98 (d, 1H), 7.20 (d, 1H), 7.63 (t, 1H).

2-methyl-6-(tributylstannyl)pyridine

2-Bromo-6-methyl pyridine (3.0 g, 17 mmol) was dissolved to THF and cooled to –78 °C. 1.6 M *n*-BuLi (11.28 mL) was slowly added to the solution and stirred for 75 min. Then tributyltin chloride (5.55 mL. 20 mmol) was slowly added to the solution and stirred for 4 h. After warming to RT, the resultant solution was extracted with Et_2O (3 times) and purified with alumina column chromatography (eluent: CH_2Cl_2). After removal of the solvent by evaporation, the yellow oil was obtained (4.25 g, 65%).
^{1}H NMR ($CDCl_3$, 300 MHz) δ/ppm: 0.89 (m, 9H), 1.10 (m, 6H), 1.32 (m, 6H) , 1.56 (m, 6H), 2.53 (s, 3H) , 6.95 (d, 1H) , 7.17 (d, 1H), 7.36 (t, 1H).

4-Carbomethoxy-6,6′-dimethyl-2,2′-bipyridine

2-methyl-6-(tributylstannyl)pyridine (1.53 g, 4 mmol) and 2-chloro-6-methylpyridine-4-carboxylic acid methyl ester (0.63 g, 3.4 mmol) were dissolved to toluene. After the addition of [Pd(PPh_3)$_4$] (200 mg), the resultant mixture was refluxed for 24 h. The resultant solution was filtered with celite and 6 M HCl (20 mL) was added to the solution. The solution was extracted with CH_2Cl_2 (3 times) and purified with alumina column chromatography (eluent: CH_2Cl_2). After removal of the solvent by evaporation, a pale yellow solid was obtained (595 mg, 71%).
^{1}H NMR ($CDCl_3$, 300 MHz) δ/ppm: 2.64 (s, 3H), 2.69 (s, 3H), 3.97 (s, 3H) , 7.18 (d, 1H), 7.69 (t, 1H) , 7.71 (s, 1H) , 8.19 (d, 1H), 8.71 (s, 1H).

6,6′-dimethyl-2,2′-bipyridine-4-carboxylic acid (L3)

4-Carbomethoxy-6,6′-dimethyl-2,2′-bipyridine (200 mg, 0.82 mmol) was dissolved to MeOH (20 mL). An aqueous solution (10 mL) of K_2CO_3 (113.6 mg, 0.82 mmol) was

added to the solution and the resultant mixture was stirred at 80 °C. After removal of MeOH by evaporation, the resultant solution was acidified to pH 3~4 with HCl aq. The white solid was obtained (170.5 mg, 90%).

^{1}H NMR ($CDCl_3$, 300 MHz) δ/ppm: 2.82 (s, 3H), 2.94 (s, 3H), 3.97 (s, 3H), 7.95 (d, 1H), 8.09 (s, 1H), 8.57 (t, 1H), 8.58 (d, 1H), 8.64 (d, 1H).

Preparation of [Cu(L3)]Cl (Y3)

6,6′-Dimethyl-2,2′-bipyridine-4-carboxylic acid (68.46 mg, 0.3 mmol) was suspended to H_2O (2 mL) and 10 drops of 1 M NaOH aq. was added to the suspension. Then an aqueous solution (1 mL) of $CuSO_4$•$5H_2O$ (37.45 mg, 0.15 mmol) was added to the resultant mixture. Further 10 drops of 1 M NaOH aq. was added to the mixture. An aqueous solution (1 mL) of ascorbic acid (30 mg, 0.17 mmol) was added to the mixture and the color of the mixture changed to reddish black. After adjusting pH 2-3 by the addition of 1 M HCl, the precipitate was obtained. The precipitate was filtered and dissolved to EtOH. After removal of the insoluble matter, the reddish-black solid was obtained by evaporation of the solvent (36 mg, 43 %).

^{1}H NMR ($CDCl_3$, 300 MHz) δ/ppm: 1.91 (s, 3H), 1.23 (s, 3H), 7.49 (d, 1H), 7.84 (s, 1H), 8.01 (t, 1H), 8.45 (d, 1H), 8.73 (s, 1H). ESI-TOF-MS *m*/*z*: 518.79 $[M-Cl]^+$ (calc. 519.11). FT-IR (solid, KBr) ν/cm^{-1}: ν 3433 (O–H), 3076, 2961, 2921, 2855 (C–H), 1718 (C=O), 1600, 1558, 1469, 1436, 1390 (C=C, C=N), δ 804, 777 (C–H). Anal. calcd. for $C_{26}H_{24}ClCuN_4O_4$·EtOH·$0.25H_2O$: C, 55.49; H, 5.07; N, 9.24. Found: C, 55.51; H, 5.14; N, 9.45.

References

1. F. Havas, N. Leygue, M. Danel, B. Mestre, C. Galaup, C. Picard, *Tetrahedron* **2009**, *65*, 7673-7686.
2. a) L. Zani, G. Reginato, A. Mordini, M. Calamante, M. Peruzzini, M. Taddei, A. Sinicropi, M. L. Parisi, F. Fabrizi de Biani, R. Basosi, A. Cavallaro, M. Bruzzi, *Tetrahedron Letters* **2013**, *54*, 3944-3948, b) D. Blanco-Ania, C. Valderas-Cortina, P. H. Hermkens, L. A. Sliedregt, H. W. Scheeren. F. P. Rutjes, *Molecules* **2010**, *15*, 2269-2301.
3. R. Grandi, W. Messerotti, U. M. Pagnoni, R. Trave, *The Journal of Organic Chemistry* **1977**, *42*, 1352-1355.
4. J. Elhaïk, C. M. Pask, C. A. Kilner, M. A. Halcrow, *Tetrahedron* **2007**, *63*, 291-298.

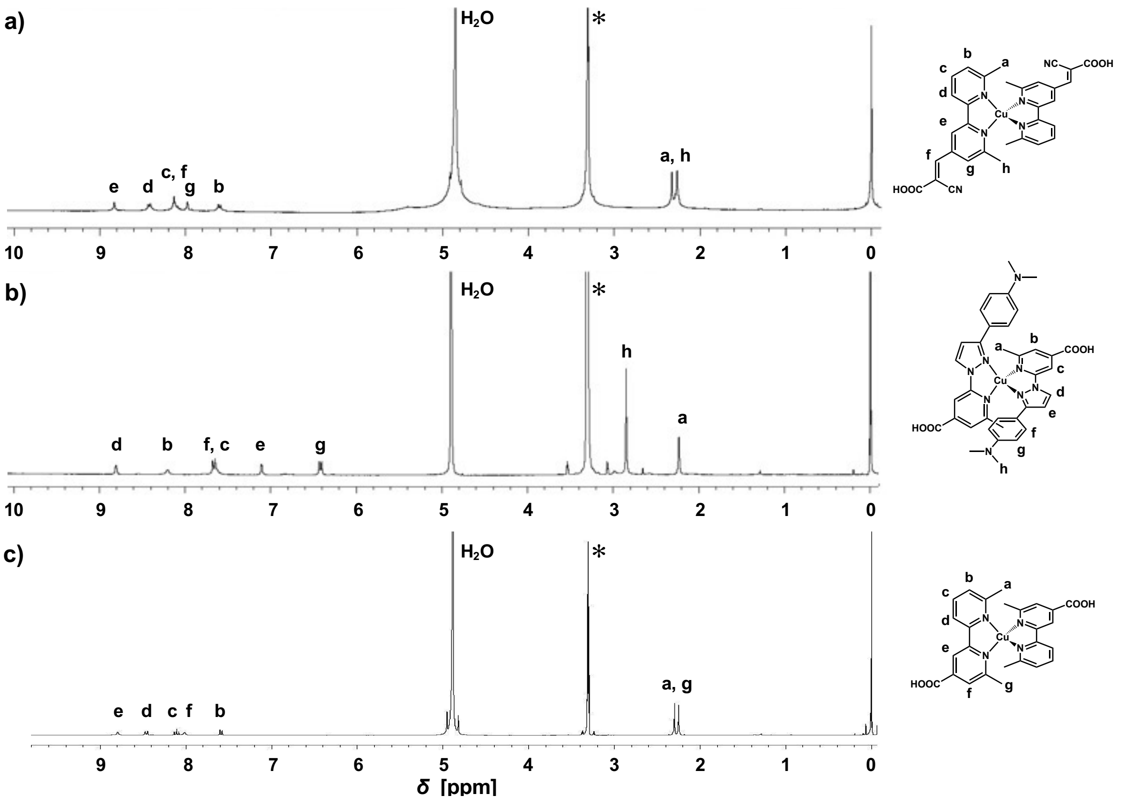

Figure S1. ^{1}H NMR spectra of a) **M1**, b) **M2**, and c) **Y3** in CD_3OD.

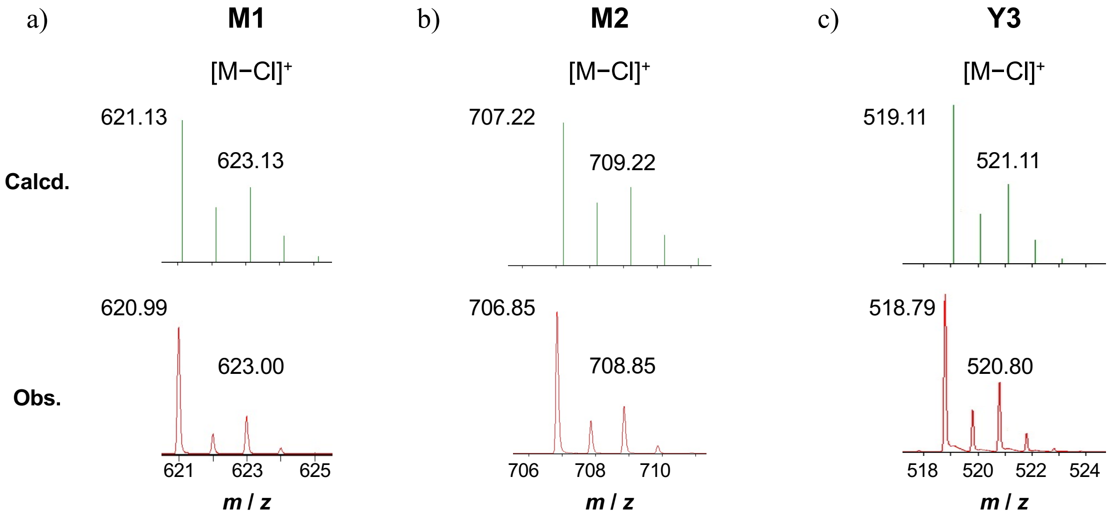

Figure S2. ESI-MS spectra of a) **M1**, b) **M2**, and c) **Y3** in MeOH (positive mode).

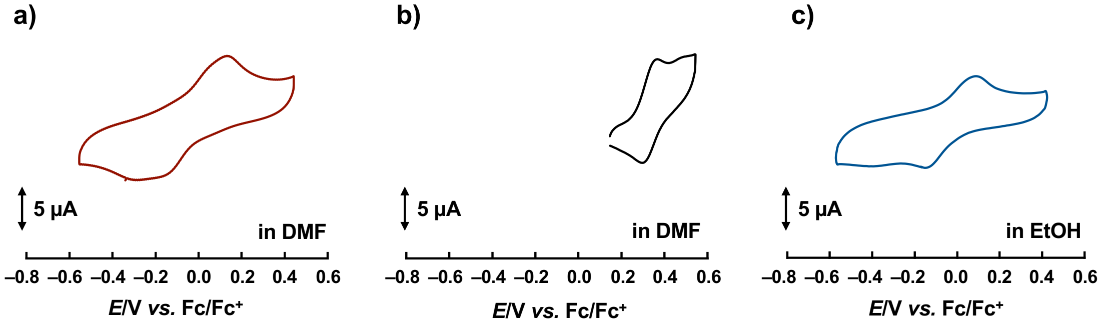

Figure S3. Cyclic voltammograms of a) **M1**, b) **M2**, and c) **Y3**. Concentration of each dye: 0.3 mM. Electrolyte solution: 0.1 M TBAP in DMF (for **M1** and **M2**) and in EtOH (for **Y3**).

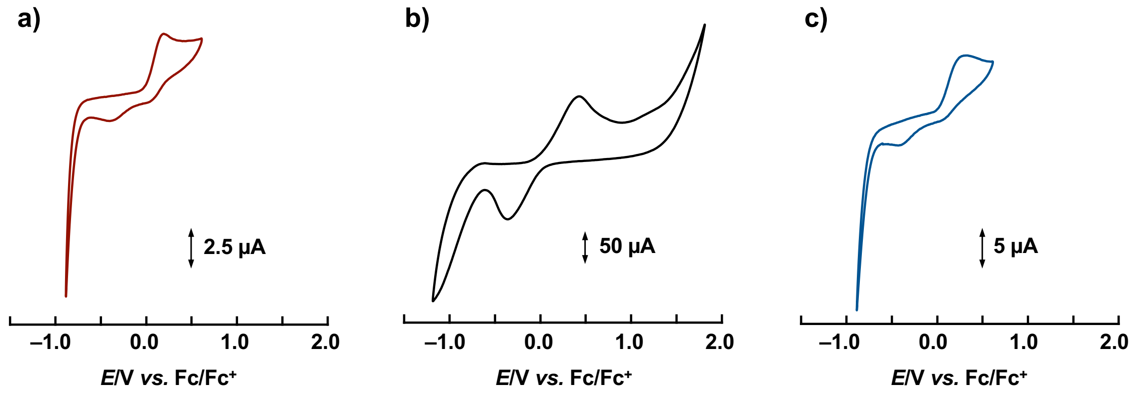

Figure S4. Cyclic voltammograms of a) **M1**, b) **M2**, and c) **Y3** on the TiO_2/FTO electrode. Electrolyte solution: 0.1 M TBAP in MeCN.

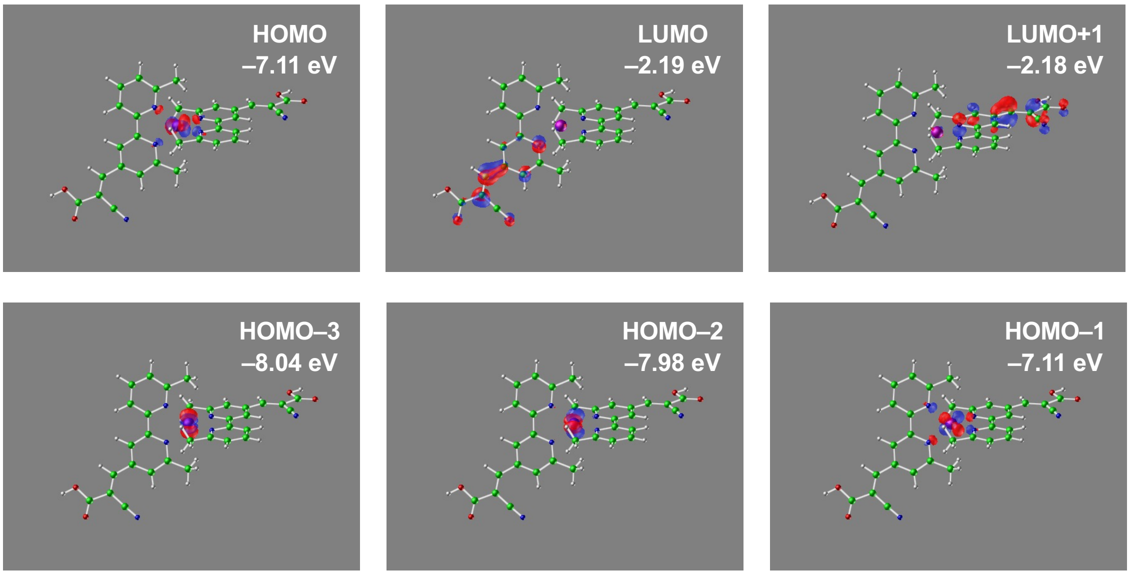

Figure S5. Isosurfaces plot of molecular orbitals of **M1** in EtOH, as calculated using CAM-B3LYP.

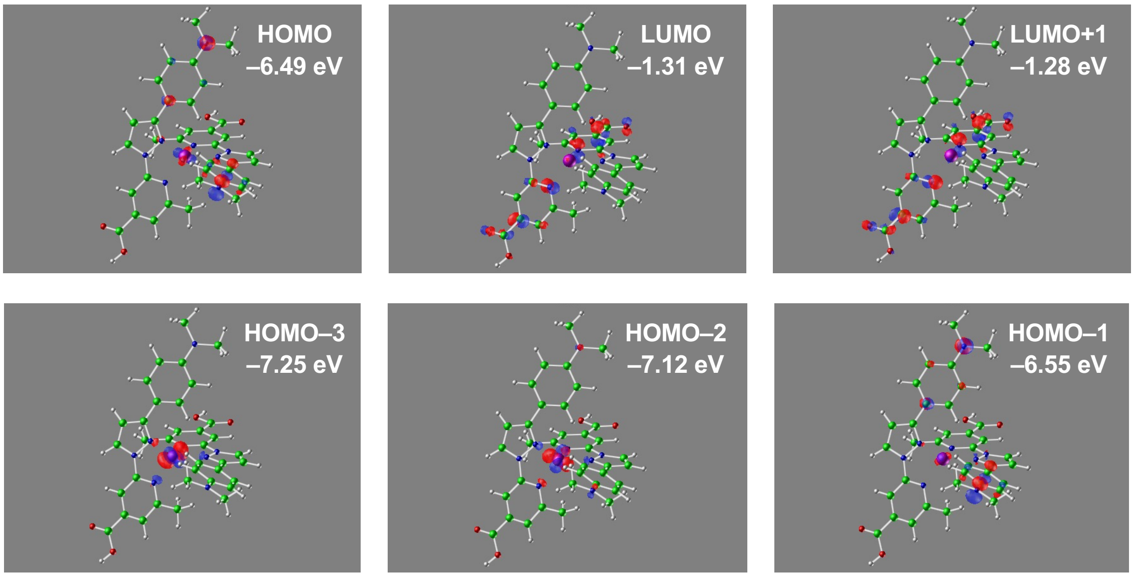

Figure S6. Isosurfaces plot of molecular orbitals of **M2** in EtOH, as calculated using CAM-B3LYP.

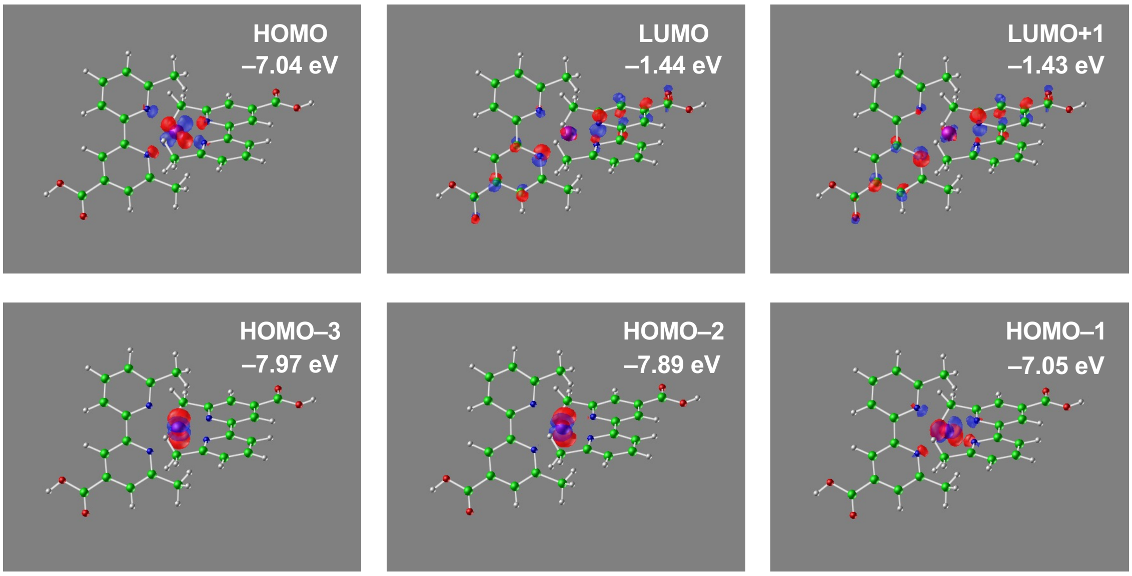

Figure S7. Isosurfaces plot of molecular orbitals of **Y3** in EtOH, as calculated using CAM-B3LYP.

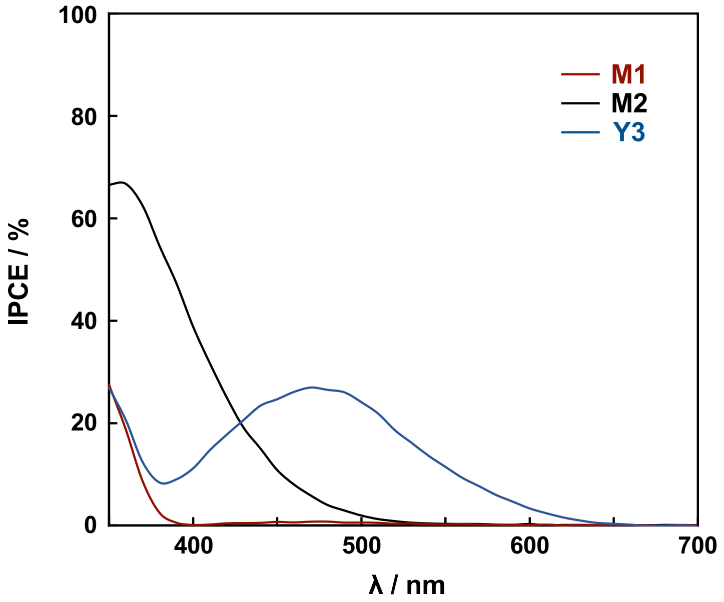

Figure S8. Incident photo-to-current conversion efficiency spectra for dye-sensitized solar cells

Table S1. Amount of each dye adsorbed on the TiO_2/FTO electrode.

Dye	Adsorption amount / μmol cm^{-2}			
	**1**	**2**	**3**	**average**
**M1**	0.13	0.11	0.09	0.11
**M2**	0.44	0.51	0.48	0.48
**Y3**	0.10	0.08	0.08	0.09
**N719**	0.07	0.09	0.07	0.08
